# Nanomedicine and graphene-based materials: advanced technologies for potential treatments of diseases in the developing nervous system

**DOI:** 10.1038/s41390-021-01681-6

**Published:** 2021-09-03

**Authors:** Giada Cellot, Audrey Franceschi Biagioni, Laura Ballerini

**Affiliations:** Neuron Physiology and Technology Lab, International School for Advanced Studies (SISSA), Trieste, Italy

## Abstract

**Abstract:**

The interest in graphene-based nanomaterials (GBNs) application in nanomedicine, in particular in neurology, steadily increased in the last decades. GBNs peculiar physical–chemical properties allow the design of innovative therapeutic tools able to manipulate biological structures with subcellular resolution. In this review, we report GBNs applications to the central nervous system (CNS) when these nanomaterials are engineered as potential therapeutics to treat brain pathologies, with a focus on those of the pediatric age. We revise the state-of-the art studies addressing the impact of GBNs in the CNS, showing that the design of GBNs with different dimensions and chemical compositions or the use of specific administration routes and doses can limit unwanted side effects, exploiting GBNs efficacy in therapeutic approaches. These features favor the development of GBNs-based multifunctional devices that may find applications in the field of precision medicine for the treatment of disorders in the developing CNS. In this framework, we address the suitability of GBNs to become successful therapeutic tools, such as drug nano-delivery vectors when being chemically decorated with pharmaceutical agents and/or other molecules to obtain a high specific targeting of the diseased area and to achieve a controlled release of active molecules.

**Impact:**

- The translational potential of graphene-based nanomaterials (GBNs) can be used for the design of novel therapeutic approaches to treat pathologies affecting the brain with a focus on the pediatric age.
- GBNs can be chemically decorated with pharmaceutical agents and molecules to obtain a highly specific targeting of the diseased site and a controlled drug release.
- The type of GBNs, the selected functionalization, the dose, and the way of administration are factors that should be considered to potentiate the therapeutic efficacy of GBNs, limiting possible side effects.
- GBNs-based multifunctional devices might find applications in the precision medicine and theranostics fields.

## Main text

Among nanomaterials, characterized by at least one dimension in the nanometer scale, one of the more interesting groups is graphene and its derivatives, named graphene-based nanomaterials (GBNs). Graphene’s chemical structure, a monolayer of carbon atoms arranged in a hexagonal matrix, confers to this material mechanical, electrochemical, and optical properties^1–3^ which, beyond other applications, favor graphene developments in biomedicine.^4^ In addition, nanomaterials match the dimensions of CNS functional units, such as dendritic spines and synaptic vesicles,^5–7^ prompting their use in neuroscience for addressing CNS function and dysfunction at nanometric resolution.^8^

In this review, we focus on engineered dispersed GBNs applications as innovative therapeutics, such as nano-carriers or drug-delivery multifunctional systems. We will briefly address the current knowledge of GBNs impact on CNS fundamental physiology, to discuss later the advantages of using chemically modified GBNs as therapeutic tools for the treatment of pediatric neuro-diseases. GBNs use as biosensors/interfaces components has been extensively reviewed elsewhere.^9,10^

We report pre-clinical results mainly in adult animal models. Indeed, GBNs treatments efficacy requires future validation in humans. In addition, new therapeutic solutions suited for adults have to be further adjusted to the pediatric population that, due to biological and/or metabolic differences, presents diverse pharmacokinetics and pharmacodynamics challenges.^11,12^ To date, owing to the fragmented pediatric market (including subjects with different degrees of development, from prenatal life to adolescence) and to the complexities in presenting pediatric clinical trials, GBNs-based nanomedicine approaches for the treatment of pediatric neuro-pathologies are still clinically unexplored.

## Not conjugated GBNs in biological milieu: their effects on nervous tissue

**Fig. 1:**
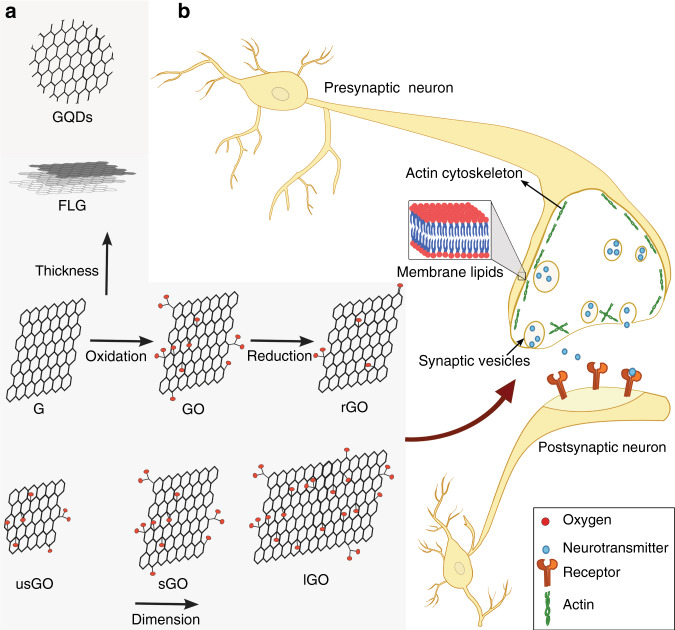
GBNs can interact with the nervous system. **a** Schematic representations of GBNs used in neuroscience studies. **b** Some of these materials modulate synaptic functions targeting active-cytoskeleton, lipid membranes, and synapse vesicles.

The family of GBNs includes few-layers graphene (FLG), graphene oxide (GO), and reduced graphene oxide (rGO), which are heterogeneous materials (sketched in Fig. 1). Physical–chemical features of GBNs and their importance are reported elsewhere.^13^

In biology applications, GBNs dispersibility in water-based solutions is crucial and depend upon GBNs chemical composition. Recent in vitro studies (summarized in Table 1) showed that pristine FLG in solution did not affect cell viability and function in cultured brain networks,^14–17^ besides being poorly dispersed and aggregating under physiological conditions.^18^ FLG, at high doses, aggregates contribute to cytotoxicity in neuronal PC12 cell lines.^19^ Conversely, oxidation, by enriching GO with oxygen-containing functional groups increases GO dispersibility in a water-based solution,^20,21^ limiting nanomaterials aggregations and favoring the interaction of GBNs with biological structures. According to this, GO nanosheets (lateral size 50–1100 nm) showed no cytotoxicity when chronically applied to human neuroblastoma SH-SY5Y cell line for four days at low doses, with only higher doses affecting cell viability.^22^

**Table 1:** Impact of GBNs on the nervous tissue.

Type of graphene	Sample or route of administration	Type of study	Treatment	GBNs impact	Refs.
lGO	Hippocampal neurons	In vitro	10 μg/mL acute	Impairment of cell viability	^14^
GO	Cortical neurons	In vitro	10 μg/mL - 3 DIV	Downregulation of glutamatergic activity and increase in GABAergic activity	^15^
sGO	Spinal cord	In vivo (zebrafish)	0.5 μg	Downregulation of glutamatergic activity	^17^
FLG	Neuronal PC12 cell lines	In vitro	>10 μg/mL	Cytotoxicity to cancer cells was improved	^19^
GO	Human neuroblastoma	In vitro	80–100 mg/mL	Dose- and time-dependent reduction of cancer cells viability	^22^
sGO	Hippocampal neurons	In vivo (rat)	50 μg/mL chronically	Transient downregulation of glutamatergic activity	^23^
sGO	Hippocampal and amygdalar neurons	In vitro	10 μg/mL acute 10 μg/mL chronically	Transient increase of glutamatergic activity Transient downregulation of glutamatergic activity	^14,16,23^
rGO	Cortical neurons	In vitro	20 μg/mL chronically	Downregulation of glutamatergic activity	^24^
lGO	Cortical neurons	In vitro	10 μg/mL - 3 DIV	Astrocyte–neuron communication was improved	^25^
sGO	Amygdalar neurons	In vitro in vivo (rat)	50 μg/mL acute	Prevented LTP and PTSD-related behavior	^31^
rGO	Olfactory bulb	In vivo (mouse)	0.004 μg/mL; long-term impact 7–21 days	Lack of cytotoxicity and impact on de novo neurogenesis	^32^
GO	Central striatum injection	In vivo (mouse)	1 μg/μL	Prevent glial cell reactivity	^23,33^
GO-188Rhenium	Tail vein injection	In vivo (mouse)	1 mg/kg	Crossing of the BBB	^37^
rGO	Tail vein injection		7 mg/kg	Transient opening of BBB	^39^
GO-porphyrin	Human brain microvascular endothelial cells	In vitro	50 μg/mL acute	Lateral size-dependent crossing of the BBB	^40^
lGO sGO usGO	Intranasal instillation	In vivo (mouse)	30 μg acute	Lateral size-dependent crossing of the BBB	^41^
small or large rGO	Oral administration (gavage)	In vivo (mouse)	60 mg/kg per 5 days	Transient impairment of neuromuscular coordination	^42^
rGO, G-COOH and G-OH Aminated (G-NH2) graphene	Neuroblastoma	In vitro	0.1–10 μg/mL	Transient toxicity to cancer cells Persistent toxicity	^50^
rGO-PEG	Astrocytes cells	In vitro In vivo (rat)	100 μg/mL 7 mg/kg, i.v.	Cytotoxicity to cancer cells was enhanced Transient BBB opening	^60^

GBNs ability to interface subcellular compartments of neurons was has been reported in in vitro studies adopting GO with different lateral dimensions.^14^ While GO flakes with large lateral dimension (lGO, >1000 nm) were toxic and reduced neuronal viability, those with small lateral size (<500 nm, sGO) synaptic activity without affecting cell viability.^14,16,23^ sGO flakes locally applied neurons in vitro, induced a transient alteration in excitatory synapses activity, without affecting inhibitory ones,^16,23^ with downregulation of glutamatergic transmission.^14,23^

Mechanistically, sGO synaptic modulation^14,16,23^ has not been completely elucidated, however, experimental data suggested that sGO interfere with the recycling of glutamatergic vesicles at the presynaptic terminals.

This modification induced by the nanomaterial was confirmed also by two independent research groups in cortical cultures.^15,24^ In the first research, the phenomenon was coupled with a modified composition of the lipid components of membranes and a small increase of the inhibitory postsynaptic activity, a difference that might depend on the larger GO used (100–1500 nm),^15^ and/or on the observed modified astrocyte–neuron communication induced by the nanomaterial.^25^

In the second work, in vitro cortical neurons were treated chronically with rGO (which presents a lower amount of oxygen-containing groups with respect to GO). The authors showed that rGO was internalized by neurons, where the nanomaterial was oxidized via cellular reactive oxygen species to GO and then found to affect the actin cytoskeleton downregulating the activity of excitatory synapses.^24^

The effects of GBNs were studied also on glial cells in vitro. Both FLG and GO did not affect astrocytes’ viability upon chronic exposures.^14,26^ However, GO and FLG (lateral size 100–1500 nm) were found to induce changes in the proteomic and lipidomic profiles of membranes, affecting astrocytes intracellular calcium dynamics.^26^

In another work, sGO increased the shedding of micro-vesicles from astrocytic membranes, a vectorized glial signaling.^14^ Thus, the GBNs mediated modulation of neuronal activity might depend on a direct effect of the nanomaterial on synapses together with a changed astrocytes-neurons communication due to downstream effects of GO on glial functionality.

## Challenges in translating not conjugated GBNs in therapeutics

The ability of sGO in downregulating excitatory synaptic transmission hints at the exploitation of this nanomaterial as a therapeutic tool to target glutamatergic synapses, developing novel treatments for neuropsychiatric disorders whose pathology is linked to an exceeding glutamatergic signaling. For instance, tumor,^27^ Tourette’s and attention-deficit/hyperactivity disorders, autism,^28^ obsessive-compulsive disorder,^29^ seizures, and epilepsy^30^ are some of the pathologies of childhood related to glutamatergic dysregulation.

The translation of GBNs in therapy requires several steps, preliminarily the validation of the effect of the nanomaterial in vivo and later the optimization of the best dose and way for administering the therapeutic.

Recent observations showed that also in vivo sGO dampen excitatory signaling in the CNS. Stereotactic injection of sGO nanoflakes into the hippocampus of juvenile rats transiently (48 h) decreased glutamatergic signaling which recovered 72 h after the treatment.^23^ This reversibility is an interesting property to exploit sGO as therapeutic tools.

In zebrafish larvae, injection of sGO into the spinal cord induced a decrease in the swimming performance; further in vivo electrophysiological recordings confirmed that sGO downregulated the release of glutamate from presynaptic terminals, affecting fictive swimming of paralyzed animals.^17^

sGO ability to target glutamatergic synapses, to transiently modulate the activity of excitatory synapses, with high spatial resolution, might be used as a therapeutic tool to interrupt dysfunctional glutamatergic activity observed in pathological conditions, as recently reported in ref. ^31^.

In this work, the authors used a rat behavioral model of post-traumatic stress disorder (PTSD). The anxiety-related behaviors, that were due to the long-term potentiation (LTP) of glutamatergic synapses in the lateral amygdala, could be rescued by a single stereotactic injection of sGO, delivering the nanomaterials precisely to this nucleus of the brain during the consolidation of the pathological plastic changes. In vitro experiments confirmed that an exposure to sGO could prevent the LTP of amygdalar excitatory synapses,^31^ suggesting sGO ability to interrupt the dis-functional plasticity leading to PTSD.

Among GBNs, also rGO were tested in vivo. After intracranial injection in the mouse olfactory bulb, rGO was biocompatible, and did not induce post-injection apoptosis, or impaired de novo neurogenesis, a relevant feature of this brain region.^32^

Regarding the GBNs impact on glial cells in vivo, sGO directly injected into rat hippocampus reduced the gliotic and neuroinflammatory responses usually observed upon surgery^23^ displaying lower tissue reactivity with respect to the saline vehicle,^33^ suggesting that GO exerted a protective effect against the tissue damage.

GBNs intracranial brain injection is an invasive procedure. This method, targeting specific brain areas, avoids systemic toxicity,^34^ but is justified only for pathological conditions with no effective cure or requiring comparably invasive treatments (such as the surgical procedures used to treat pharmacologically refractory forms of epilepsy^35^). However, other ways of GBNs administration seem successful in targeting the CNS. Bio-distribution profiles, after rodent tail vein injection of GBNs, showed the accumulation of nanomaterials in several organs including the brain.^36,37^ In detail, GO (lateral dimension 10–800 nm) radiolabeled with 188Rhenium were traced in the brain from 1 until 48 h after injection, suggesting that GO 37 crossed the blood–brain barrier (BBB), which protects the encephalon.^38^ Similarly, the presence of rGO after tail vein injection (7 mg/kg) was detected using mass-spectrometry imaging throughout diencephalon,^39^. The authors proposed that rGO favor transient BBB openings, assessed by the expression of junctional proteins.

Indeed, Su and collaborators showed the BBB crossing of GO nanosheets (lateral size 20–500 nm) in an in vitro model composed of human brain microvascular endothelia cells.^40^ In addition, they reported that changes in the lateral size of GO might influence the degree of permeability of the nanomaterial once conjugated with a therapeutic: larger nanosheets appearing to cross more than smaller ones, with no apparent cytotoxic effect. Alternative routes for a less invasive GBNs administration to the CNS are the nasal and oral ones. A recent study demonstrated that GO with different lateral dimension (lGO, sGO, and ultrasmall-, usGO), upon delivery into the nasal cavities translocated to the brain with us-GO achieving the wider distribution, with GO size affecting BBB crossing. GO presence declined in 1 month, consistently with biodegradation.^41^

Upon oral administration at high doses, rGO with small and large lateral dimensions (80 and 500 nm) could be detected in a very low amounts in the brain. The treated animals, analyzed through MRI and histology, did not show CNS morphological alterations, but expressed reversible impairment in neuromuscular coordination in the first days of treatment (but fully recovered by 15 days), which was ascribed to a general discomfort of the animals owing to a large amount of rGO retained in the body rather than to a toxic effect on the nervous system.^42^

In vivo CNS impact of GBNs was reported also in other works,^43–47^ but we did not include these here as they focused on the environmental effects of nanomaterials due to large-scale GBNs productions, more than on biomedical applications, or since the GBNs physical–chemical properties were not specified.^48^

A summary of GBNs effects on the nervous system is reported in Table 1. We will discuss in the next paragraph how GBNs can be tailored by modifying their chemical structure to target the CNS as nanovectors for gene, protein, and drug-delivery aimed to treat pathologies bypassing BBB impermeability.

## GBNs as multifunctional drug-delivery system for the nervous system

**Fig. 2:**
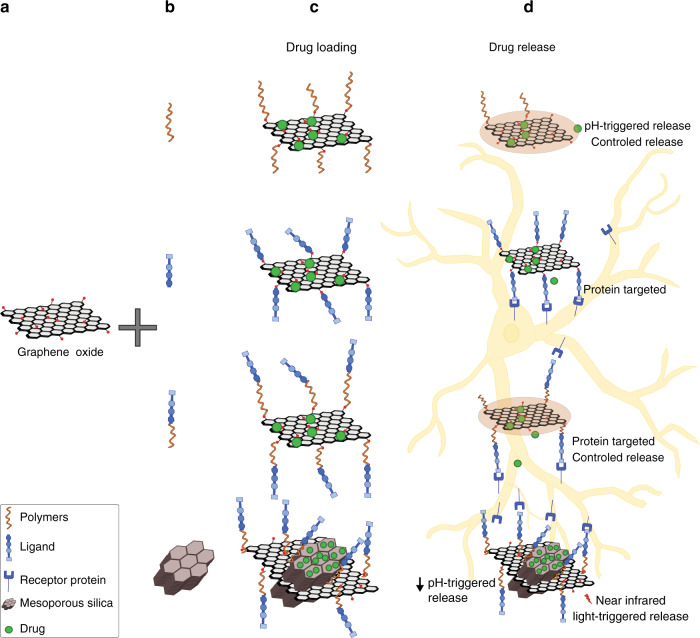
Modified GBNs as systems to deliver therapeutics and/or biomolecules in the CNS. The chemical structure of some GBNs, such as GO (**a**), is suited to be decorated with polymers, biomolecules, or mesoporous materials (**b**) to improve the loading of drugs (**c**) and their release (**d**) to specific targets and/or in a controlled manner.

Current research on target-delivery exploits GBNs as nano-carriers. GBNs large surface area allows chemical modification by adding hydroxyl, carboxyl, amino, or other functional groups.^49^ Although these functionalized GBNs have been reported to exert toxic effects also at low doses on cultured human neuroblastoma cells,^50^ likely due to increased reactivity of the modified nanomaterial with the subcellular components, such chemical structures are fundamental to bind additional molecules to improve the biocompatibility and hydrophilicity properties of the GBNs. Figure 2 and Table 2 summarize the chemistry used to link active biomolecules^51^ for delivery systems. Alternatively, GBNs can be loaded with therapeutic agents via noncovalent functionalization (hydrophobic or π–π stacking interactions and hydrogen bonding).^52^ Research efforts were mostly directed to new brain cancers therapies, due to limitations in current therapeutic approaches, such as the poor hydrophilicity of many anticancer agents, the reduced accessibility of the CNS, and the unspecific toxicity of chemotherapy on healthy tissue.^53^ Thus, GBNs were exploited as nanocarrier to treat CNS tumors that show a high mortality rate among children.^54^ The current use of aggressive treatments increases the survival rate in some cases,^55^ however, late- and long-term effects of childhood cancer treatment can be severe and irreversible leading to secondary neoplasms, emotional disorders, and cognitive dysfunctions.^56–58^ Graphene as drug-delivery nanocarrier holds promises for childhood and adult neuro-oncology.

**Table 2:** GBNs for drug-delivery to the CNS.

GBNs-based systems	Loaded drug	Disease	Model	Treatment	Activity	Refs.
GO-PEG	SN38	Glioma	In vitro	50 nM; 72 h of incubation	Increase hydrophilicity and cytotoxicity on cancer cells	^59^
GO-PAA	1,3-bis(2-chloroethyl)-1-nitrosourea	Glioma	In vitro	0–100 µg/mL	Improve solubility and dispersibility/retain drug activity/prolongate the half-life	^61^
GO-Transferrin-PEG	Doxorubicin	Glioma	In vitro In vivo (rat)	3 µg/mL 2 mg/Kg (tail vein injection)	Faster uptake rate/selectively accumulated in the tumor region/reduced glioma volume/increased lifespans	^64^
GO-lactoferrin	Puerarin	Parkinson’s disease	In vitro In vivo (mouse)	1–50 μM 5 mg/kg (tail vein injection)	Crossing of BBB/reduced behavioral responses related to Parkinson’s disease	^65^
GO–PDEA	Camptothecin	Glioma	In vitro	0.1–100,000 nM	Release of drug at reduced pH/cytotoxicity against glioma was enhanced	^67^
GO-mesoporous silica /IL13r	Doxorubicin	Glioma	In vitro	50 µg/mL	Specific target release/reduced pH- and photo-thermal stimulation-dependent release/cytotoxicity against glioma was enhanced	^68^
GO-polyamidoamine	Epirubicin/Let-7g miRNA	Glioma	In vitro In vivo (mouse)	1.3 µg/mL/16.3 nM (tail vein injection)	Specificity and cytotoxicity of drugs were improved/reduced pH-dependent release/selectively release at tumor tissue	^73^
GQDs	Clitoria ternatea	Alzheimer’s disease	In vivo (rat)	3 mg/kg	Improved learning and memory capacity	^76^
GQDs	Doxorubicin	Glioblastoma cells	In vitro	200–250 µg/mL; 24 h of incubation	Changing membrane permeabilization/cytotoxicity of cancer cells/synergism between GQDs and Dox	^77, 78^
rGO-Fas	Sevoflurane	Focal cerebral ischemia	In vivo (rat)	5 mg/kg (intravenous injection)	Specific target release/inhibition of neuronal degeneration	^82^
GQDs	–	Parkinson’s disease	In vivo (mouse)	50 µg/50 mL (chronical intraperitoneal injection)	Crossing of BBB/inhibition of alpha-synuclein	^87^
GQDs	–	Alzheimer’s disease	In vitro	10 mM	Electrostatic interaction with peptides/inhibition of amyloid-B 1–42 peptide	^89^
GO	–	Alzheimer’s disease	In vivo (mouse)	0.03 g/kg (intranasal administration)	Cognitive memory deficits were ameliorated and brain glial activation were reduced	^92^

An example of GBNs as vector for anticancer drug was reported by Liu and collaborators,^59^ who observed that by functionalizing GO (lateral size < 50 nm) with polyethylene glycol (PEG) the hydrophilicity and biocompatibility were enhanced in respect to pristine GO. Plus, the modified material adsorbs easily different types of insoluble anticancer drugs, such as camptothecin (CPT, a widely used water-insoluble anticancer drug) and SN38. These authors demonstrated that SN38 not covalently bound to the GO-PEG complexes was stable in solution for several days exerting pharmacological activity on U87MG glioma cells in vitro.^59^ Conversely, when the PEGylated nanomaterial was rGO, the resulting conjugate exerted toxic effects on astrocytes both in vitro and in vivo, while the non-PEGylated rGO did not induce cytotoxicity.^60^. In a different study, after being modified with polyacrylicacid (PAA), GO were covalently bound to 1,3-bis(2-chloroethyl)-1-nitrosourea (BCNU), a commercial chemotherapeutic drug used against brain tumors. Once tested on GL261 glioma cell line, BCNU covalently linked to the graphene-based carrier showed to retain 70% of the drug activity and to have a prolonged half-life in respect to that of not conjugated molecule. This work showed that GBNs may deliver drugs or biomolecules controlling their release and customizing the pharmacokinetics of drugs for healing CNS disorders.^61,62^

One of the main issues related to chemotherapy is the unspecific toxicity on healthy tissue, which recently was addressed by new drug-design strategies, including delivering chemotherapeutic agents to molecular targets overexpressed on the surface of tumor cells.^63^ The chemical structure of GBNs, characterized by a wide surface modifiable with multiple functional groups, is ideal to design systems combining chemotherapeutics with molecules driving the system toward the locus of the cancer.

Liu and collaborators engineered a nano-vector composed by GO (lateral dimensions of 100–400 nm, complexed with PEG to improve its solubility) linked to transferrin (Tf), a glycoprotein that binds specific receptors overexpressed at the surface of glioma cells.^64^ Then, the anticancer drug doxorubicin was loaded to the GO complex (Tf-PEG-GO-doxorubicin) and tested in vitro and in vivo.^64^ First, in vitro tests detected that Tf-PEG-GO-doxorubicin was uptaken by C6 glioma cells at a faster rate when compared to unconjugated doxorubicin or GO-doxorubicin. Moreover, when administered in brain glioma-bearing rats through tail vein injections, the drug-loaded GO complex accumulated selectively in the region of the tumor, reducing glioma volume and increasing rats lifespans. This suggested a significant improvement in the therapeutic efficacy of doxorubicin when complexed in the GBNs nanoscale system.^64^

A similar strategy was successful also when targeting the Parkinson’s disease murine model, where GO-based nanovectors (lateral size of ~250 nm) complexed with lactoferrin were used to increase BBB crossing and targeting of the diseased tissue, characterized by an overexpression of lactoferrin receptors.^65^

An alternative approach to deliver a chemotherapeutic specifically to cancer cells exploits the typical acid pH detected in cancer tissue as a signal to activate drug release^66^ and was exploited by Kavitha and co-workers,^67^ who functionalized covalently GO with a pH-sensitive smart polymer (poly(2-(diethylamino) ethyl methacrylate)), PDEA. Then CPT was adsorbed on the GO–PDEA complex and its efficacy was tested in vitro on N2a neuronal cancer cells. The authors observed that when the nano-system was exposed to low pH (mimicking tumor conditions) it released CPT, a release not detected at physiological pH. CPT complexed to PDEA-induced cytotoxicity of cancer cells in vitro more efficiently than the pure drug, while the GO–PDEA system (without CPT) did not exert toxic effects per se.^67^

Chemotherapeutic efficacy can be improved in GBNs systems by combining different strategies, such as the targeted delivery and the pH-sensitive release of the drug. An example of this was reported by Wang et al.^68^, who used mesoporous silica-coated GO nanosheets (lateral size 50–250 nm) modified with a peptide able to bind the receptor of interleukin 13 (IL13r), overexpressed in some malignant tumors, including glioma. This system was loaded through adsorption with doxorubicin. The authors demonstrated that thanks to the presence of the mesoporous silica, the system was efficient in releasing doxorubicin in a pH-related manner (more release in an acid environment). In addition, since graphene is a photosensitizer with high absorption in the near-infrared wavelength, the pH-dependent release of doxorubicin could be enhanced by stimulating photo-thermally the nanomaterial.

The therapeutic effect of doxorubicin containing nanodevices was demonstrated in six hours long-lasting incubations on U251 human glioma cultured cells in terms of an increased cytotoxicity when compared to that induced by the nanodevice without the peptide binding the IL13r. To note, the same treatment on 1800 human astrocyte (healthy) cells did not show changes in their viability. Moreover, the photo-thermal stimulation of the doxorubicin containing nanodevices boosted the cytotoxicity of U251 human glioma. Since a decrease in cell viability upon photo-thermal stimulation was observed also in glioma cells treated with not doxorubicin-loaded devices, this suggested that such nanodevices might benefit from a synergetic effect of the chemo- and thermal-therapy.^68^ This system, allowing a targeted and controlled release of doxorubicin, might contribute to limit the severe side effects of this drug, which include cardiotoxicity in childhood cancer.^69^

GBNs are also ideal candidates for designing innovative tools in theranostics^70^ to develop devices integrating therapy to diagnostics. These systems can deliver a drug and at the same time allow to localize the targeted cells and to quantify the amount of therapeutic reaching the target. This is achievable, for instance, by exploiting the intrinsic imaging properties of GBNs^71^ or conjugating the nanomaterial with a detectable agent.^72^

Yang and collaborators,^73^ designed a complex graphene-based system for theranostic applications. After modifying GO with a low molecular weight polymer (polyamidoamine) to increase compatibility with physiological solutions, the nanomaterial was loaded with two therapeutics: the anticancer drug epirubicin (EPI) and the micro RNA Let-7g (miRNA), known to downregulate the expression of Ras oncogenes.^74^ Moreover, GO was bound with gadolinium (Gd), a magnetic resonance imaging (MRI) agent, allowing to detect the nano-complex in bioimaging. The system transferred both EPI and Let-7g miRNA into glioma U87 cells in vitro, inducing respectively DNA disruption and knocking-down the Ras family proteins expression. Once administered systematically in vivo through tail vein injection, the GBNs-based nano-systems were imaged in the brain through MRI. Although in this study the GBNs could reach the brain thanks to a focused ultrasound-induced permeabilization of the BBB, these results appear promising for the potential use of GBNs-based systems carrying therapeutic together with imaging molecules.^73^

An alternative approach for the treatment of brain cancer refers to the use of graphene quantum dots (GQDs). Thanks to their extremely reduced size (<20 nm) and to their ability to cross the BBB,^75^ they are ideal as drug-delivery platforms. Tak and collaborators reported that the modification of GQDs surface chemistry varied the biological effect of the nanomaterials on nervous tissue.^76^ U87 glioma cells treated with doxorubicin presented an enhanced decrease in cell viability if pretreated with GQDs, Green-GQDs, or COOH-GQDs respect to cells exposed to the chemotherapeutic only. Differently, the pretreatment with NH2-GQDs did not exert modifications in the effect of doxorubicin.^77,78^ This was due to the differential impact of the modified GQDs on cell membrane permeability: cells treated with Green-GQDs or COOH-GQDs, but not those with NH2-GQDs, exhibited an increased membrane fluidity, that correlated to the surface net charges of the GQDs and favored cellular uptake of the chemotherapeutic drug.^78^

Many studies focused on the impact of GBNs in anticancer-related drug-delivery systems,^79,80^ but the high ability of adsorbing/binding molecules enables GO to load and release drugs for several neurologic diseases. Pediatric stroke is a rare condition, but it can cause severe long-lasting disabilities in the majority of affected children.^81^ The design of a GBNs-based system to deliver in the locus of the ischemic brain a neuroprotective agent was reported by Wu and collaborators.^82^ The authors used rGO (lateral size of 100 nm) conjugated with a ligand of Fas, a transmembrane protein whose expression was found to increase after the onset of a stroke^83^ to address the nanocarrier toward the site of the lesion. This system was loaded with sevoflurane (SF), a neuroprotective agent that decreases the inflammation in the cerebral infarct.^84^ In an animal model of focal cerebral ischemia, the nano-complex, thanks to the presence of the Fas ligand, distributed more significantly to the ischemic brain region, instead of being randomly distributed across the entire brain.^82^ The efficacy of SF linked to the nanocarrier in inhibiting neuronal degeneration was tested in vivo, indicating that the SF (at a concentration of 5 mg/kg) complexed to the nano-system was more efficient than the traditional SF (10 mg/kg) treatment.^82^ Thus, the conjugation of GBNs with antibodies or other ligands appears a potential strategy to improve the delivery of neuroprotective drugs for the treatment of cerebral ischemia.

Another possible application of GBNs falls within the field of pediatric neurological conditions that are associated with abnormal protein aggregation, such as hemimegalencephaly, tuberous sclerosis complex, and focal cortical dysplasia. These pathologies were recently reported to be characterized by upregulated levels or even pathological aggregation of abnormally phosphorylated Tau protein.^85^ Although no studies on the effect of GBNs on these neurodevelopmental diseases are available, we briefly reported here that GO, in conjugation with quantum dots (GQDs) were used for the treatment of adulthood abnormal CNS protein aggregations, such as those found in Parkinson’s and Alzheimer’s diseases.^86^

Using a Parkinson’s disease animal model, it was shown that GQDs (with dimensions ≈5–20 nm) upon chronic intraperitoneal injections crossed the BBB and inhibited the aggregation of alpha-synuclein,^87^ a presynaptic neuronal protein, involved in neuronal death.^88^

Similarly, GQDs (diameters ≈ 8 nm) also inhibited deposition of the Amyloid-β (Aβ)1–42 peptide in vitro,^89^ which is a key factor for the development of Alzheimer’s disease.^90^ The inhibition in the Aβ peptides aggregation had a positive correlation with the decrease of the QDGs surface negative charge, suggesting a possible electrostatic interaction.^89^ Also GQDs (diameters ≈ 8 nm) produced through a green synthesis starting from Clitoria ternatea demonstrated that the nanomaterial, after being injected in an animal model of Alzheimer’s disease, was able to ameliorate the typical deficit in learning and memory.^76^

In addition, thanks to their ability to chelate metals which are involved in the formation of Amyloid-β peptides aggregates, GO were proposed as an alternative strategy for the treatment of Alzheimer’s disease.^91^ When loaded with the anti-inflammatory agent dauricine, and administered nasally to mice models of Alzheimer’s disease, GO could ameliorate cognitive deficits and reduce glial activation by combining the effects of the nanomaterial and the drug.^92^

Thus, graphene-based drugs may be suitable tools for the therapy of abnormal protein aggregation observed also in pediatric age diseases.

## Conclusions

Thanks to their nanoscale dimensions, GBNs can be engineer to influence CNS functions. GO is the most interesting GBNs, due to its ability to modulate neuronal activity per se, without further functionalization, while upon proper chemical modifications it can be exploited into graphene-based complex drug-delivery systems, allowing to target specific CNS site for controlled release of the pharmaceutical agents. This paves the way toward their use in the field of precision medicine.

In this framework, GBNs potential toxicity has been actively explored. The design and manufacturing of the GBNs, their size, functionalization, dose, and way of administration are all factors that allow limiting possible side effects. Although promising, the efficacy of GBNs-based treatments will require to be validated in the clinic, and specifically in the pediatric population.
